# Crosslinked agarose-gelatine beads as a substrate for investigating biofilms of bacterial pathogens

**DOI:** 10.1016/j.bioflm.2026.100380

**Published:** 2026-07-08

**Authors:** Dan Roizman, Maren Herzog, Arpita Nath, Nivetha Pachaimuthu, Ahmad Hujeirat, Benno Kuropka, Jens Rolff, Alexandro Rodríguez-Rojas

**Affiliations:** a Evolutionary Biology, Institut für Biologie, Freie Universität Berlin, Berlin, Germany; b Clinical Department for Small Animals and Horses, Unit of Small Animal Internal Medicine, University of Veterinary Medicine, Vienna, Austria; c Institute of Chemistry and Biochemistry, Freie Universität Berlin, Berlin, Germany

## Abstract

Treating chronic bacterial infections remains challenging because biofilm formation reduces bacterial susceptibility to antimicrobial agents. However, many *in vitro* biofilm models rely on abiotic surfaces that poorly reflect host-associated environments. Here, we developed a crosslinked agarose-gelatine hydrogel substrate in the form of autoclavable beads for biofilm cultivation. *Escherichia coli*, *Pseudomonas aeruginosa* and *Staphylococcus aureus* rapidly colonised the beads and formed significantly greater biofilm biomass than on glass beads. Quantitative proteomic analyses revealed substrate-dependent physiological differences, including increased abundance of several virulence-associated proteins in biofilms grown on agarose-gelatine beads. In addition, the hydrogel matrix functioned as a reservoir for quorum-sensing molecules and diffusible pigments. Together, these findings indicate that agarose-gelatine beads provide a practical and physiologically relevant platform for investigating biofilm-associated bacterial phenotypes beyond conventional abiotic surfaces.

## 1 Introduction

Biofilms are microbial communities embedded in a self-produced extracellular matrix that facilitates adhesion and surface colonisation [1,2]. They occur widely across natural and host-associated environments [3], and biofilm growth is considered a predominant mode of microbial life on Earth [4]. Bacterial biofilms are strongly associated with chronic infections, medical device-associated infections, and treatment failure due to their reduced susceptibility to antimicrobial agents [[5], [6], [7]]. Despite extensive research efforts, biofilm cultivation methods remain highly diverse and often lack physiological relevance [8].

Most *in vitro* biofilm models use abiotic substrates such as glass, plastic, silicone or metal surfaces [[8], [9], [10], [11], [12]]. While these platforms are experimentally convenient and compatible with microscopy and quantitative assays, they may not accurately reflect the structural and physicochemical properties encountered by biofilms in host-associated environments [13]. These limitations highlight the need for cultivation systems that better represent biologically relevant growth conditions.

The importance of cultivation substrates has been recognised since the early development of microbiology. In 1881, Robert Koch introduced gelatine-based solid media for bacterial cultivation and diagnostics. Although gelatine was later largely replaced by agar because of its superior thermal stability and resistance to bacterial degradation [14,15], Koch's work demonstrated how cultivation substrates can influence the observation of bacterial growth and phenotype [16]. In biofilm-associated infections, bacterial physiology is similarly shaped by the surrounding matrix and growth surface. Biologically compatible hydrogel substrates may therefore provide more physiologically relevant experimental models than conventional abiotic materials [15].

Agarose-based hydrogels are widely used in biotechnology because of their biocompatibility, permeability, and ease of chemical modification [17,18]. Previous studies have used agar or agarose beads to model chronic bacterial infections and spatially structured bacterial growth, particularly in respiratory infection models involving *Pseudomonas aeruginosa* [[19], [20], [21]]. Bead-based cultivation systems have also been employed to study biofilm formation, bacterial persistence, and antimicrobial susceptibility [[22], [23], [24]]. More broadly, bacterial attachment to bead-associated surfaces has been recognised since some of the earliest investigations of surface-associated microbial growth [25,26].

Here, we integrated these conceptual and methodological approaches by developing crosslinked agarose-gelatine beads (AGBs) as a biologically compatible hydrogel substrate for biofilm cultivation. We evaluated the platform using *Escherichia coli* (*E. coli*), one of the best-characterised bacterial model organisms [27], as well as the clinically important opportunistic pathogens *Pseudomonas aeruginosa* (*P. aeruginosa*) and *Staphylococcus aureus* (*S. aureus*). These species represent diverse bacterial lineages, infection types, and biofilm lifestyles. Using this experimental framework, we compared biofilm formation on AGBs and conventional glass beads and investigated substrate-dependent differences in bacterial physiology, virulence-associated traits, and global protein expression.

## 2 Results

### 2.1 Development and physicochemical characterisation of AGBs

**Fig. 1:**
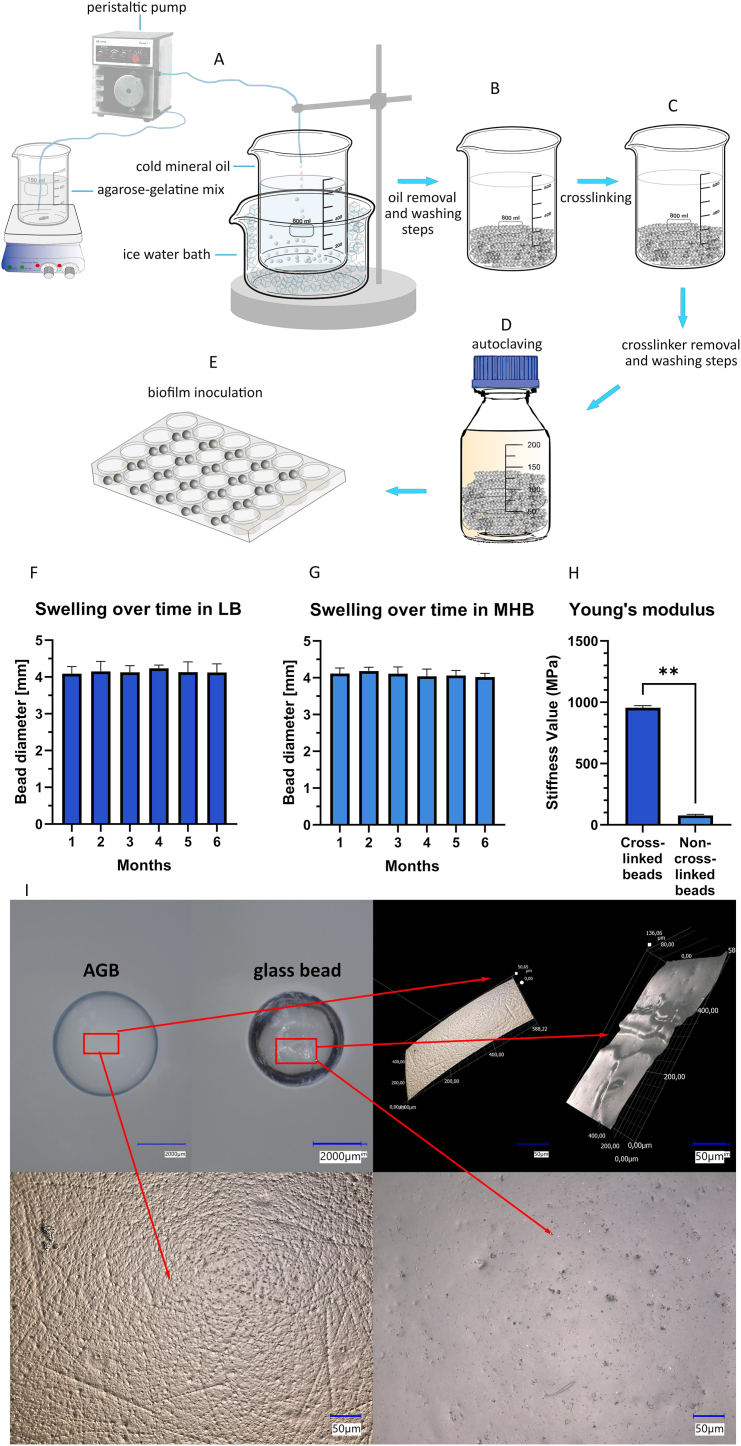
Development, fabrication workflow, and physicochemical characterisation of crosslinked AGBs. Schematic representation of the workflow used for generating crosslinked AGBs. A heated mixture containing 2% agarose and 2% gelatine was continuously dispensed into ice-cold mineral oil to generate millimetre-scale spherical beads (A). Following bead formation, residual mineral oil was removed by repeated washing steps before divinyl sulfone (DVS) crosslinking (B–C). After crosslinking and washing, the beads were autoclaved and stored until use (D). For biofilm experiments, beads were transferred into 24-well plates and inoculated with bacterial cultures (E). Long-term swelling stability of AGBs following crosslinking and autoclaving during storage in LB (F) and MHB (G) for six months. Bars represent the mean bead diameter of five beads (N = 5), and error bars represent the standard deviation. DVS crosslinking significantly increased bead stiffness, as measured by Young's modulus using atomic force microscopy (H). Statistical significance was determined using Student's t-test, p < 0.0001. Comparative surface morphology of AGBs and conventional glass beads acquired using a Keyence VHX-X1 microscope (I). Unlike the smooth surface of glass beads, AGBs exhibited a heterogeneous hydrogel architecture with increased structural complexity. Additional fabrication details are provided in the Materials and Methods section, Supplementary Protocol I, and Supplementary Video S1.

We developed a crosslinked agarose-gelatine hydrogel substrate based on two classical semisolid matrices widely used in microbiology. Using a divinyl sulphone (DVS)-mediated crosslinking strategy adapted from Porath, Låås and Janson [18], we evaluated multiple agarose, gelatine and crosslinking conditions to identify formulations suitable for generating stable spherical beads. The crosslinking of the beads using DVS is similar to the fabrication of Sepharose, a chromatographic matrix commonly used for biomolecule purification [17]. A composition containing 2% agarose and 2% gelatine produced the most reproducible and mechanically stable beads, whereas lower concentrations (0.5-1%) generated highly deformable structures and higher concentrations (3-8%) solidified too rapidly for reproducible handling. Spherical AGBs were generated by depositing the heated hydrogel mixture into ice-cold mineral oil, resulting in stable bead structures (Fig. 1A–E, Fig. S1, Supplementary Protocol I). Preliminary experiments also indicated that agarose-only beads supported reduced biofilm growth compared with AGBs, while gelatine-only beads exhibited insufficient stiffness and poor crosslinking stability.

Initially, bead generation was performed manually using a multi-stepper pipette to dispense the hot agarose-gelatine mixture into ice-cold mineral oil (Supplementary Video S2). While suitable for small-scale production, this workflow was subsequently adapted into a semi-automated high-throughput system using continuous pumping and temperature-controlled mixing to improve scalability and reproducibility (Fig. S1–S2, Supplementary Videos S1–S2, Supplementary Protocol I). This approach enabled the rapid production of highly reproducible AGBs with an average diameter of approximately 4 mm and a coefficient of variation below 2% across five independent batches (Fig. 1, Fig. S1 and Fig. S3 ). The resulting beads were comparable in size to conventional glass beads used for biofilm cultivation [28]. DVS crosslinking substantially improved bead rigidity and thermal stability, enabling autoclaving without structural degradation.

Next, we characterised the long-term stability of the beads following crosslinking and autoclaving. AGBs remained structurally stable for at least six months, with no detectable changes in bead appearance or diameter during storage in culture medium (Fig. 1F–G). Crosslinking also significantly increased bead stiffness, as demonstrated by Young's modulus measurements (Fig. 1H). In contrast to non-crosslinked hydrogels, crosslinked AGBs tolerated autoclaving while maintaining their structural integrity. Surface comparison between conventional glass beads and AGBs revealed substantial differences in texture and material architecture (Fig. 1I). Unlike the smooth, chemically inert surface of glass beads, AGBs exhibited a heterogeneous hydrogel surface with greater structural complexity, potentially providing distinct attachment and diffusion environments for bacterial biofilm formation.

Supplementary data related to this article can be found online at https://doi.org/10.1016/j.bioflm.2026.100380

The following are the Supplementary data related to this article:

Multimedia component 6

Multimedia component 7

### 2.2 *E. coli*, *P. aeruginosa* and *S. aureus* form robust biofilms on the AGBs

**Fig. 2:**
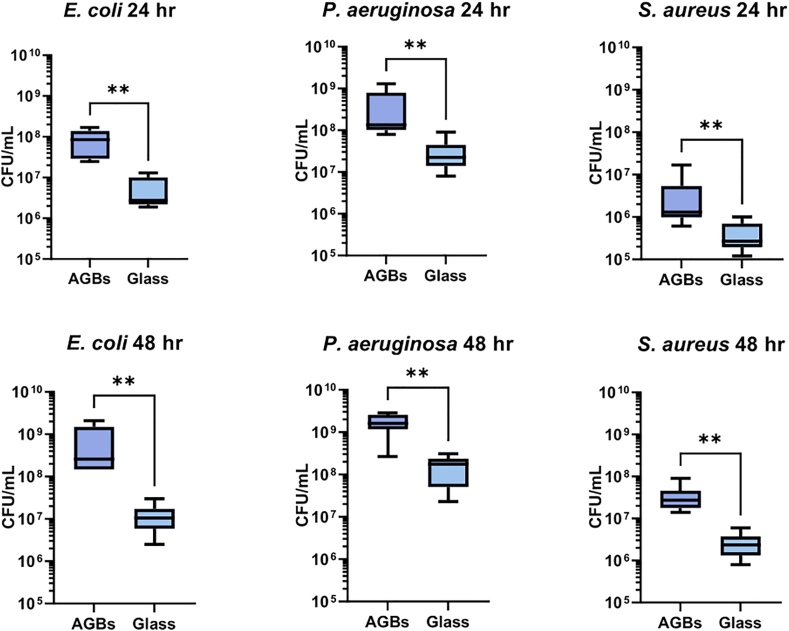
Comparative biofilm formation of *E. coli*, *P. aeruginosa*, and *S. aureus* on AGBs and glass beads after 24 and 48 h. Boxplots of bacterial counts from six biofilm-containing beads for each species grown on the surface of the beads at 24 and 48 h (N = 6). Samples were compared using the Wilcoxon-Mann-Whitney test, and two asterisks indicate p-values <0.01. Boxes extend from the 25th to the 75th percentiles, whiskers represent the minimal and maximal values, and the middle line represents the mean.

*E. coli*, *P. aeruginosa* and *S. aureus* formed mature biofilms on the surfaces of AGBs, and these biofilms were compared with those on conventional glass beads. Quantification of viable biofilm-associated cells by colony-forming units (CFU) revealed significantly greater biofilm biomass on AGBs than on glass beads for all three bacterial species at both 24 and 48 h of biofilm maturation (Fig. 2).

Because recovery of bacterial cells from hydrogel-associated biofilms was initially more variable than from glass beads, we optimised the dispersion protocol by combining mechanical disruption with enzymatic digestion using DNase I and papain (see Materials and Methods and Supplementary Methods). This approach improved biofilm dispersion and recovery of single bacterial cells without affecting viability, enabling reliable CFU quantification of AGB-associated biofilms.

### 2.3 AGBs are compatible with fluorescence and scanning electron microscopy

**Fig. 3:**
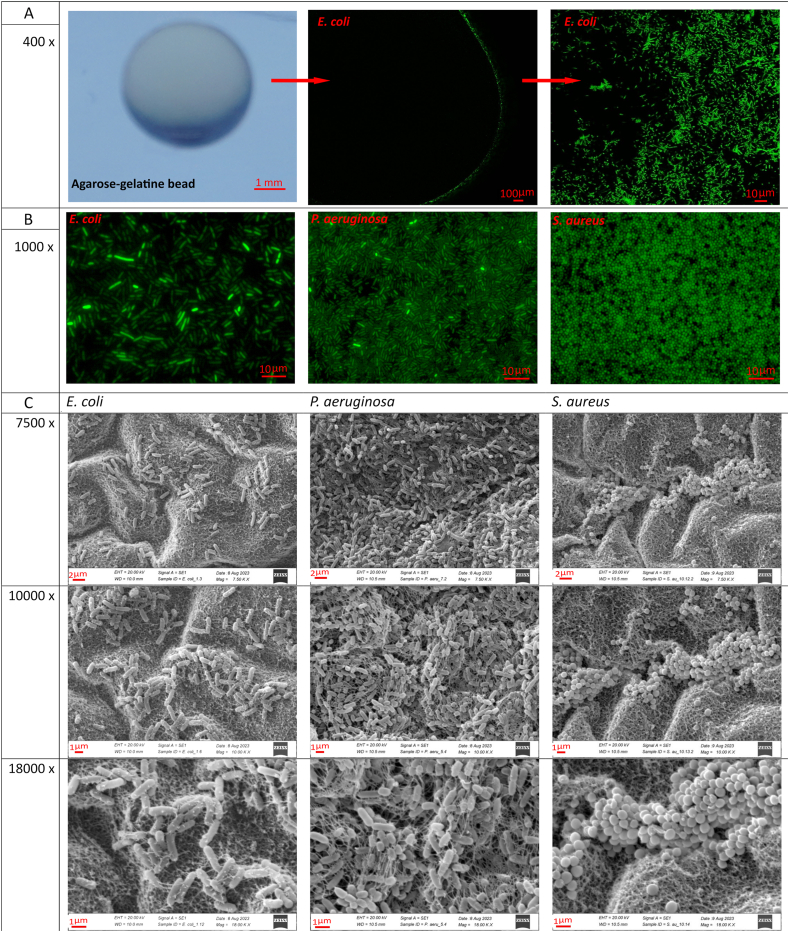
Microscopy of AGB biofilms using fluorescence microscopy, confocal laser scanning microscopy, and scanning electron microscopy (SEM). GFP-labelled *E. coli*, *P. aeruginosa*, and *S. aureus* were cultivated on AGBs for 48 h to establish mature biofilms (A). Confocal Z-stack imaging comprising 29 optical sections revealed dense surface-associated *E. coli* biofilm formation on the AGB surface, while a reconstructed cross-sectional image demonstrated uniform biofilm coverage of the bead. To facilitate stable microscopy observation of 4 mm spherical AGBs, a custom 3D-printed adapter (B) was developed to simplify imaging and ensure compatibility with oil-immersion objectives (see Materials and Methods and Fig. S4). Following optimisation of fixation and dehydration procedures, AGB-associated biofilms of *E. coli*, *S. aureus*, and *P. aeruginosa* were visualised using SEM at multiple magnifications after 48 h of cultivation (C).

The transparency of AGBs enabled direct visualisation of biofilm-associated bacterial cells using live fluorescence and confocal laser scanning microscopy. To evaluate imaging compatibility, GFP-labelled *E. coli*, *P. aeruginosa* and *S. aureus* biofilms were cultivated on the surface of AGBs and visualised by fluorescence microscopy and confocal imaging (Fig. 3A). Confocal Z-stack imaging revealed dense surface-associated biofilm formation and uniform coating of the AGB surface by *E. coli* biofilms. To facilitate stable microscopy observation of 4 mm spherical AGBs, we developed a custom 3D-printed adapter (Fig. 3B, Fig. S4), which enables simplified handling, stable bead positioning during imaging and compatibility with oil-immersion objectives.

AGB-associated biofilms were also compatible with scanning electron microscopy (SEM). Because of the hydrogel structure and spherical morphology of the beads, optimisation of fixation, dehydration and drying procedures was required to preserve both bead integrity and biofilm architecture during SEM sample preparation (see Materials and Methods). The best structural preservation was achieved using critical point drying, enabling high-resolution SEM imaging of *E. coli*, *S. aureus* and *P. aeruginosa* biofilms formed on the surface of the AGBs after 48 h of cultivation (Fig. 3C). The agarose-gelatine matrix maintained sufficient structural stability throughout sample processing to allow detailed visualisation of biofilm-associated cellular organisation and surface architecture.

### 2.4 Quantitative proteomics reveals substrate-dependent physiological differences between biofilms formed on AGBs and glass beads

**Fig. 4:**
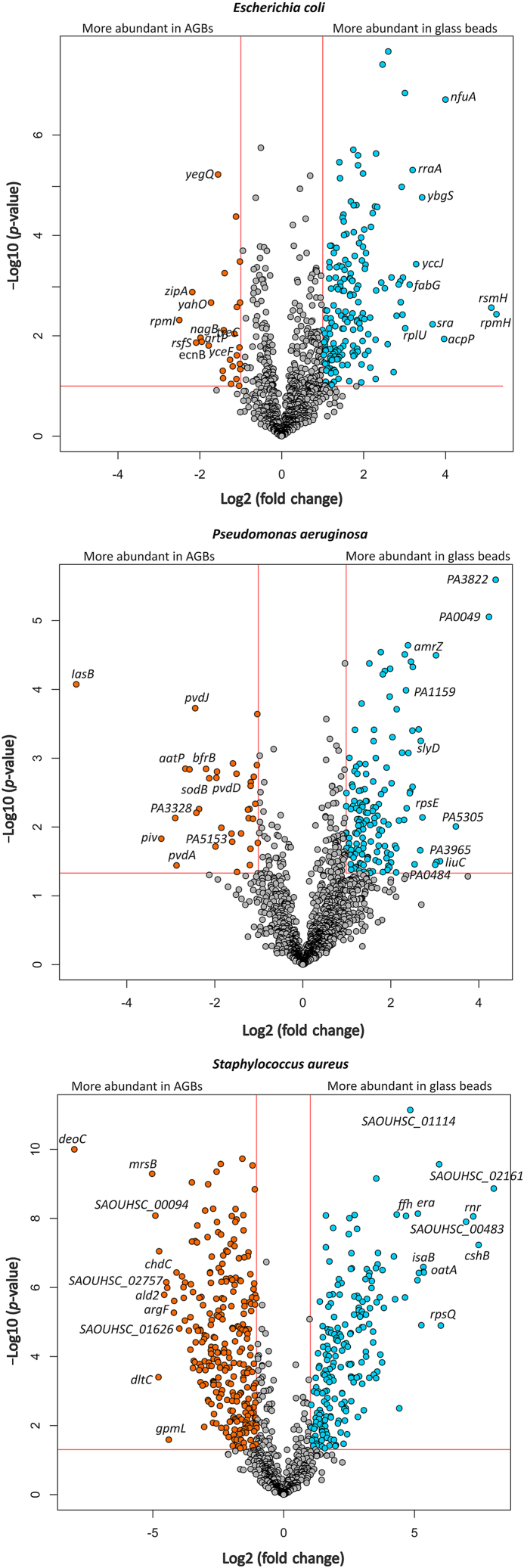
Comparison of relative proteome-wide expression between agarose-gelatine beads and glass beads for bacterial biofilms of *E. coli*, *P. aeruginosa*, and *S. aureus*. Volcano plots show relative changes in protein intensity between AGBs and glass beads, measured by LC-MS and label-free quantification. Orange dots denote proteins significantly more abundant in AGB biofilms, while blue dots indicate proteins more abundant in glass-bead biofilms. Proteins were considered significantly changed if they showed at least a twofold difference in relative abundance (log2 fold change >1 or < −1) with an FDR-adjusted p-value <0.05, as indicated by the orange threshold lines. (For interpretation of the references to colour in this figure legend, the reader is referred to the Web version of this article.)

Label-free quantitative proteomics (liquid chromatography-mass spectrometry, LC-MS) revealed distinct substrate-dependent protein expression profiles in biofilms formed on AGBs compared with those formed on glass beads for *E. coli*, *P. aeruginosa* and *S. aureus*. Using a modified proteomic workflow adapted for this study [29,30], we identified and quantified 1113 proteins in *E. coli*, 1500 in *P. aeruginosa* and 1251 in *S. aureus* (Supplementary Tables S1–S3). Proteins showing at least a two-fold difference in relative abundance between biofilms formed on glass beads and AGBs, together with an FDR-adjusted *p*-value <0.05, were considered significantly differentially abundant. Across all three species, biofilms formed on AGBs and glass beads exhibited distinct substrate-dependent physiological signatures (Fig. 4).

### 2.5 *E. coli* biofilms exhibit substrate-dependent differences in ribosomal regulation and cell division dynamics

In *E. coli* biofilms, the most pronounced substrate-dependent differences were associated with ribosomal regulation, translational control and cell division dynamics. Biofilms formed on AGBs showed increased abundance of several proteins associated with stationary-phase adaptation and ribosomal restructuring, including the 50S ribosomal subunit protein L35 (RpmI; log2FC = 2.5), the 30S ribosomal subunit protein S16 (RpsP) and the ribosomal silencing factor RsfS. RsfS is typically associated with slow-growing or mature bacterial populations and inhibits translation through ribosomal silencing [31], suggesting that AGB-associated biofilms may adopt a more mature physiological state.

In contrast, biofilms formed on glass beads exhibited an increased abundance of multiple ribosome-associated proteins involved in translational regulation, ribosome maturation, and stress adaptation, including RpmH, RplU, RsmH, Sra, YgaM, Rmf, and NusA. Among these, RpmH displayed the strongest differential abundance on glass beads (log2FC = −5.2). Several of these proteins are associated with ribosomal biogenesis, stationary-phase adaptation, ribosome dimerisation and stress-responsive translational regulation [[31], [32], [33]], collectively indicating a distinct translational and regulatory state in biofilms formed on abiotic glass surfaces.

Additional differences were observed in proteins associated with bacterial cell division. Biofilms formed on AGBs showed increased abundance of ZipA (log2FC = 2.2), a membrane-anchoring protein required for FtsZ Z-ring stabilisation. In contrast, glass-bead biofilms exhibited increased abundance of ZapA (log2FC = −2.1), another regulator of FtsZ polymerisation and divisome organisation [34]. Together, these findings reveal substrate-dependent differences in translational regulation and cell division-associated physiology during *E. coli* biofilm maturation.

### 2.6 *P. aeruginosa* biofilms formed on AGBs exhibit increased abundance of virulence-associated and quorum-sensing-regulated proteins

In *P. aeruginosa*, biofilms formed on AGBs exhibited increased abundance of several extracellular virulence-associated proteins and quorum-sensing-regulated factors compared with biofilms formed on glass beads. The most strongly enriched protein on AGBs was the elastase LasB (log2FC = 5.1), a major extracellular protease regulated by the LasR/LasI quorum-sensing system [35]. Additional secreted proteases involved in host interaction and extracellular protein degradation, including PrpL (Protease IV; log2FC = 3.2) and the aminopeptidase Lap/PaAP, were also more abundant in AGB-associated biofilms. Together, these results demonstrate increased abundance of extracellular virulence-associated proteins in biofilms formed on the hydrogel substrate [36].

AGB-associated biofilms also exhibited increased abundance of multiple proteins involved in pyoverdine biosynthesis and iron acquisition, including PvdA, PvdJ, PvdD and PvdL. Pyoverdine is the major siderophore produced by *P. aeruginosa* and plays an important role in iron acquisition during host-associated growth and infection [37]. The increased abundance of pyoverdine-associated proteins further supports the presence of substrate-dependent physiological differences between biofilms formed on AGBs and on glass beads.

In contrast, biofilms formed on glass beads exhibited increased abundance of several proteins associated with stress adaptation, membrane-associated regulation and translational control. Among these were the transcriptional regulator AmrZ/AlgZ (log2FC = 3.0), which regulates alginate production and motility [38,39], together with components of the Sec translocon system, including YajC (log2FC = −4.4) and SecF (log2FC = −2.5). Several highly abundant proteins in glass-bead biofilms were also annotated as putative, hypothetical or uncharacterised proteins, including PA0049 and PA5305. Notably, PA0049 expression is associated with the stringent response regulator DksA in *P. aeruginosa* [40]. Overall, these findings indicate that AGB-associated biofilms favour extracellular virulence-associated and iron-acquisition physiology, whereas biofilms formed on glass beads exhibit a broader stress-associated and regulatory adaptive response.

### 2.7 *S. aureus* biofilms exhibit extensive substrate-dependent regulatory and virulence-associated physiological restructuring

*S. aureus* exhibited the largest substrate-dependent proteomic shift among the three bacterial species analysed, with 247 proteins showing increased abundance in AGB-associated biofilms and 297 proteins enriched in glass-bead biofilms. Compared with *E. coli* and *P. aeruginosa*, *S. aureus* displayed substantially broader restructuring of regulatory, metabolic, stress-response and virulence-associated pathways, indicating pronounced physiological adaptation to the underlying substrate.

Biofilms formed on AGBs exhibited increased abundance of several proteins associated with metabolic adaptation, oxidative stress tolerance and mature biofilm physiology. Among the most strongly enriched proteins were DeoC (log2FC = 5.0) and additional components of the deo operon, including DeoB and DeoD, which are involved in nucleoside metabolism [41,42]. AGB-associated biofilms also showed increased abundance of the methionine sulfoxide reductase MsrB (log2FC = 4.9), an oxidative stress-protective enzyme associated with mature biofilms [43]. Similarly, DltC, which contributes to teichoic acid biosynthesis, cell envelope charge regulation, and virulence-associated cell surface properties, was also found to be more abundant [44].

Several regulatory proteins enriched in AGB-associated biofilms were associated with anaerobic metabolism, quorum sensing, and stress adaptation, including ArcR, CodY, SrrA, NusA, NusB, NusG, RsbW, and VraR. These regulators are associated with arginine metabolism, nutrient-responsive virulence regulation, low-oxygen adaptation, transcriptional control and cell-wall stress responses [[45], [46], [47], [48], [49], [50], [51], [52], [53], [54], [55]]. In addition, AGB-associated biofilms exhibited increased abundance of quorum-sensing-associated proteins such as TraP and LuxS, together with virulence-associated factors including LipA, KatA and components of the type VII secretion system (EsxA and EsaG), indicating enhanced density-dependent and host-associated virulence physiology on the hydrogel substrate [56,57].

In contrast, biofilms formed on glass beads exhibited increased abundance of several proteins associated with alternative virulence strategies, stress-responsive regulation, and surface-associated adaptation. Among the most abundant proteins on glass beads were SAOUHSC_02161, a putative MHC class II analogue protein regulated by the SaeRS two-component system [55,58], together with multiple transcriptional regulators, including SarR, FapR, Rex, RecX, MsrR and Spx. These regulators are associated with redox balance, lipid homeostasis, DNA damage responses, oxidative stress adaptation and the regulation of agr-dependent virulence pathways [[45], [46], [47], [48], [49]]. Glass-bead biofilms also exhibited increased abundance of proteins involved in staphyloxanthin biosynthesis (AldH1, CrtM, CrtP and CrtN), together with fibrinogen-binding proteins, leukocidin-like proteins, phenol-soluble modulins, coagulase-associated proteins and multiple staphylococcal antigens, indicating extensive restructuring of extracellular virulence-associated physiology on abiotic glass surfaces.

Overall, the proteomic profiles of *S. aureus* revealed distinct physiological and regulatory states associated with biofilm growth on AGBs and glass beads. While AGB-associated biofilms exhibited signatures consistent with mature, metabolically adapted and quorum-sensing-associated physiology, biofilms formed on glass beads displayed broader stress-associated and surface-adaptive virulence responses.

### 2.8 AGBs enable the detection of biofilm-deficient phenotypes

**Fig. 5:**
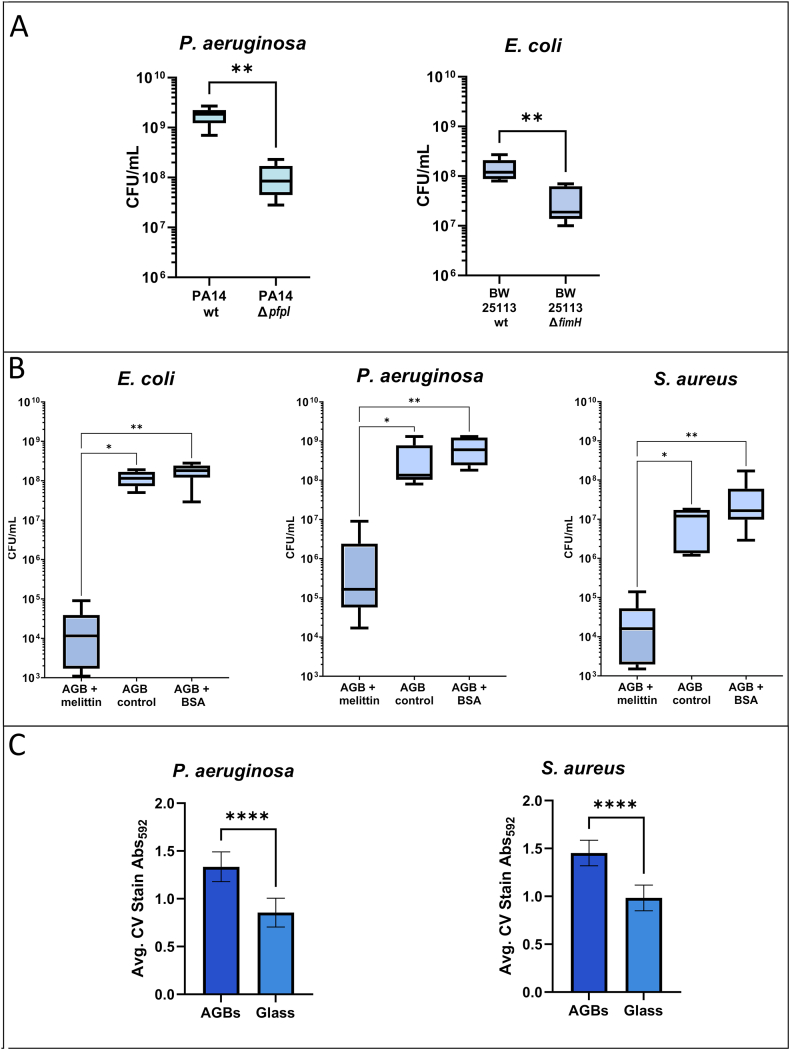
Diverse applications of AGBs for investigating biofilm formation, anti-biofilm surface modification, and crystal violet-based biofilm quantification. (A) Biofilm-deficient strains of *E. coli* (ΔfimH) and *P. aeruginosa* (ΔpfpI) were cultivated on AGBs after 24 and 48 h. Boxplots show bacterial counts from mutant biofilms compared with their corresponding parental wild-type strains (N = 6). (B) Comparative biofilm formation of *E. coli*, *P. aeruginosa*, and *S. aureus* on melittin-functionalised AGBs after 48 h of incubation. Boxplots show bacterial counts from biofilms attached to melittin-coupled, uncoupled, and BSA-coupled beads (N = 6). (C) Biofilm formation of *P. aeruginosa* and *S. aureus* on AGBs and glass beads after 48 h of incubation was quantified using the modified crystal violet staining protocol. Bar charts show the average absorbance at 592 nm of crystal violet retained by the biofilm biomass, which serves as a semi-quantitative measure of biofilm formation. Ten replicates were analysed for each species-substrate combination (N = 10). Samples were compared using the Wilcoxon-Mann-Whitney test. One asterisk indicates p < 0.05, two p < 0.01, three p < 0.001, and four p < 0.0001. (For interpretation of the references to colour in this figure legend, the reader is referred to the Web version of this article.)

To evaluate whether AGBs could discriminate between strains with distinct biofilm-forming capacities, we examined two previously characterised biofilm-deficient mutants alongside their corresponding parental wild-type strains. In *E. coli*, we analysed a mutant defective in type I fimbriae-mediated attachment, a process that contributes to submerged biofilm formation and initial surface colonisation via the FimH adhesin [59]. In *P. aeruginosa*, we examined disruption of the *pfpI* gene, which has previously been associated with impaired biofilm formation [60]. Cultivation of both mutant strains on AGBs resulted in significantly reduced biofilm formation compared with their respective wild-type strains (Fig. 5A). These findings demonstrate that the AGB platform can reliably detect substrate-dependent differences in biofilm formation and discriminate biofilm-deficient phenotypes under standard cultivation conditions.

### 2.9 Chemical functionalisation of AGBs enables investigation of anti-biofilm surface modifications

To evaluate whether AGBs could serve as a chemically modifiable platform for investigating anti-biofilm surface treatments, we functionalised the bead surface with melittin, an antimicrobial peptide previously reported to exhibit anti-biofilm activity [61]. Taking advantage of the chemical reactivity of crosslinked agarose, melittin was covalently coupled to AGBs via cyanogen bromide activation, following the protocol described by March et al. [62].

Biofilm cultivation on melittin-functionalised AGBs resulted in a substantial reduction in bacterial attachment compared with untreated control beads and bovine serum albumin (BSA)-coupled control beads (Fig. 5B). Across all three bacterial species examined (*E. coli*, *P. aeruginosa* and *S. aureus*), biofilm-associated bacterial counts decreased by approximately 4-5 orders of magnitude relative to the controls. In contrast, BSA-coupled beads exhibited the highest levels of biofilm formation, indicating that the coupling chemistry itself did not account for the observed inhibition.

Final planktonic bacterial densities remained comparable across all experimental conditions, suggesting that the effects of melittin functionalisation were primarily associated with inhibition of surface attachment and biofilm development rather than general bacterial killing. Together, these findings demonstrate that AGBs can serve as a chemically programmable platform for investigating anti-biofilm surface modifications and biofilm-material interactions.

### 2.10 AGB biofilms are compatible with crystal violet-based biofilm quantification

Crystal violet staining is one of the most widely used methods for the rapid quantification of bacterial biofilm biomass [63]. To evaluate whether this assay could be adapted for AGB-associated biofilms, we compared crystal violet staining of biofilms formed on AGBs and conventional glass beads.

Application of standard crystal violet protocols to AGBs initially resulted in increased background stain retention relative to glass beads, likely reflecting differences in the hydrogel surface properties of the substrate. The assay was subsequently optimised for AGB-associated biofilms (see Materials and Methods).

Following optimisation, crystal violet quantification confirmed that biofilms formed on AGBs accumulated greater biomass than biofilms formed on glass beads across the tested bacterial species (Fig. 5C). These findings demonstrate that conventional crystal violet-based biofilm assays can be successfully adapted for hydrogel-associated biofilms cultivated on AGBs.

### 2.11 AGBs retain diffusible bacterial metabolites and quorum-sensing molecules

**Fig. 6:**
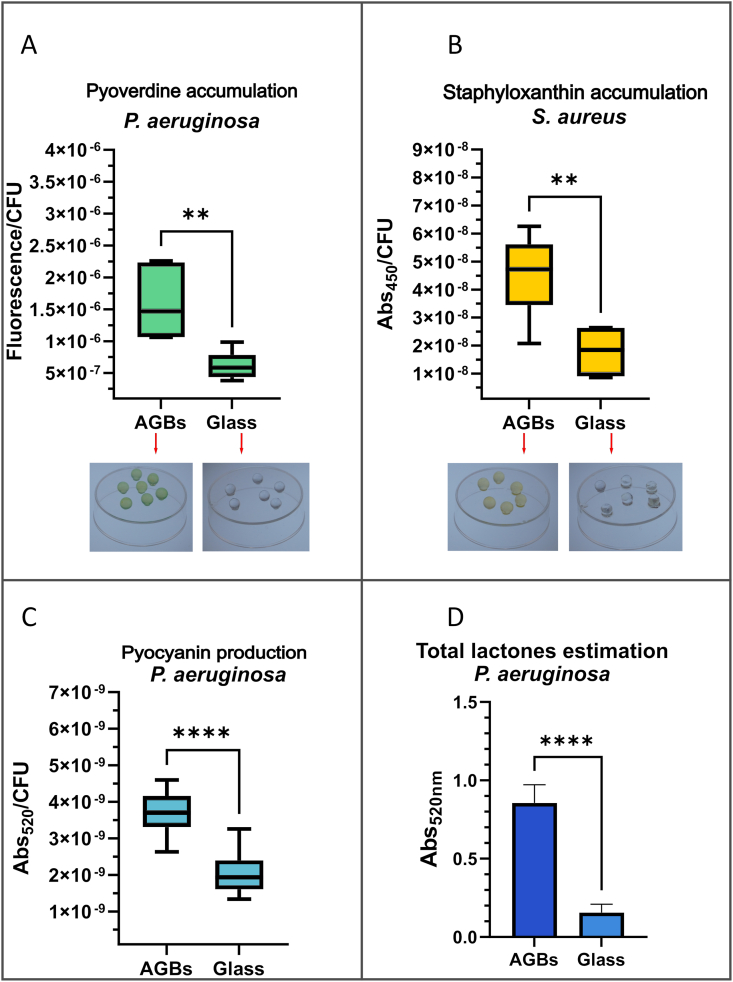
Comparative quantification of virulence-associated metabolites produced by P. aeruginosa and S. aureus biofilms grown on AGBs and glass beads after 48 h of incubation. Boxplots show accumulation of (A) pyoverdine, (B) staphyloxanthin, and (C) pyocyanin from biofilm cultures after 48 h of incubation (N = 6). (D) Quantification of N-acyl homoserine lactones (AHLs) in *P. aeruginosa* cultures indicates possible retention of diffusible quorum-sensing molecules within AGB-associated biofilm systems. Samples were compared using the Wilcoxon-Mann-Whitney test. One asterisk indicates p < 0.05, two p < 0.01, three p < 0.001, and four p < 0.0001. Boxes extend from the 25th to the 75th percentiles, whiskers represent the minimal and maximal values, and the middle line represents the mean.

During biofilm cultivation, AGBs accumulated visible bacterial pigments within the hydrogel matrix, indicating diffusion of soluble bacterial metabolites into the beads' internal structure. Biofilms formed by *S. aureus* produced progressive yellow-orange colouration of the AGBs, whereas *P. aeruginosa* biofilms generated a characteristic green pigmentation throughout the bead structure (Fig. 6A–B). These observations suggested that the hydrogel matrix could act as a local reservoir for diffusible bacterial products.

Proteomic analysis indicated substrate-dependent differences in pigment-associated pathways between the two species. In *P. aeruginosa*, proteins associated with pyoverdine biosynthesis were enriched in AGB-associated biofilms. In contrast, proteins involved in staphyloxanthin biosynthesis were more abundant in *S. aureus* biofilms formed on glass beads. To further investigate pigment accumulation in bead-containing cultures, we adapted extraction protocols to quantify bacterial pigments relative to CFU counts spectrophotometrically (see Materials and Methods). Quantitative analysis confirmed differential accumulation of pigment-associated metabolites in AGB-containing biofilm cultures (Fig. 6A–C), consistent with recent related approaches for pigment extraction and quantification [64]. We next investigated whether AGBs could also retain bacterial quorum-sensing molecules. Using a modified colourimetric assay [65], with additional modifications, we quantified N-acyl-homoserine lactones (AHLs) in *P. aeruginosa* PAO1 biofilm cultures. We observed significantly higher AHL accumulation in AGB-associated samples than in glass-bead cultures (Fig. 6D). Together, these findings indicate that the hydrogel matrix can retain diffusible bacterial metabolites and signalling molecules, supporting the concept that AGBs may partially mimic the chemically retentive microenvironments encountered in host-associated biofilms.

## 3 Discussion

**Fig. 7:**
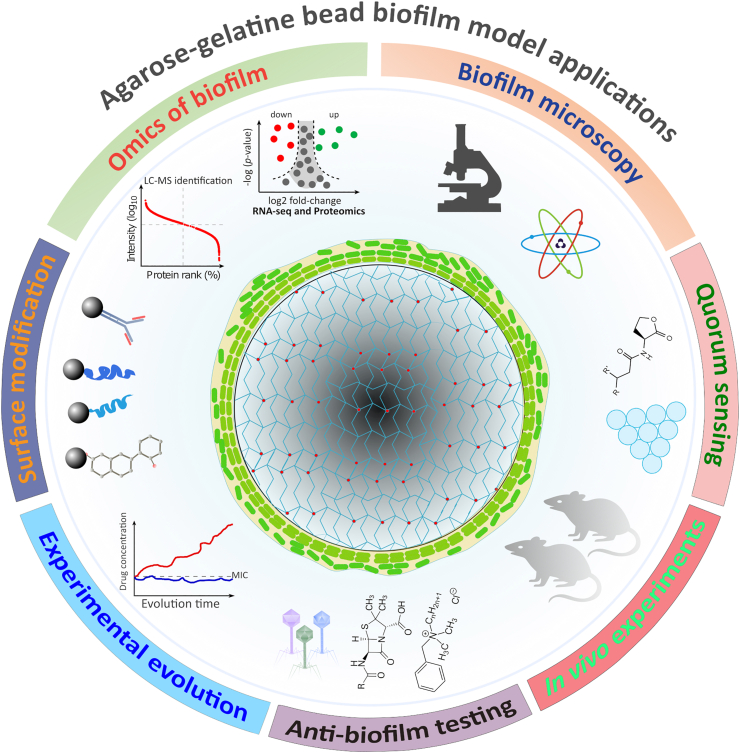
Summary of the potential applications of the crosslinked agarose-gelatine bead model system. The biocompatibility, structural stability, and versatile physicochemical properties of AGBs provide a flexible platform for biofilm-related research and applications. Their adaptability to nutrient-rich and nutrient-limited environments enables investigation of biofilm physiology under diverse experimental conditions. AGBs can be used to investigate anti-biofilm compounds, evaluate antibacterial treatments and surface coatings, and study quorum-sensing mechanisms within biofilms. Furthermore, the beads are compatible with multiple analytical approaches, including live fluorescence microscopy, confocal microscopy, and scanning electron microscopy.

The AGB platform developed in this study provides a biologically compatible and experimentally versatile substrate for investigating bacterial biofilms (Fig. 7). Compared with conventional abiotic substrates such as glass beads, AGBs support robust biofilm formation by *E. coli*, *P. aeruginosa*, and *S. aureus* while remaining compatible with established biofilm methodologies, including fluorescence and confocal microscopy, scanning electron microscopy, crystal violet quantification, viable-cell enumeration, and quantitative proteomics. Together, these features establish AGBs as a practical hydrogel-based platform for investigating biofilm physiology and biofilm-material interactions. The development of biofilm models that more closely reproduce host-associated environments has become a growing focus of biofilm research, reflecting a growing recognition that conventional abiotic systems may not fully capture clinically relevant biofilm behaviour [13].

One important feature of the AGB system is the chemical reactivity of the crosslinked hydrogel matrix, which enables covalent functionalisation of the bead surface. Using the antimicrobial peptide melittin as a proof-of-principle anti-biofilm compound, we demonstrated that chemical modification of AGBs can strongly inhibit bacterial surface colonisation without substantially affecting planktonic growth. This capability extends the platform's utility beyond passive biofilm cultivation, and it supports its application in investigating anti-biofilm coatings, surface-protective compounds, bioactive materials, and factors influencing bacterial attachment to hydrated surfaces [[66], [67], [68]].

In addition to supporting biofilm growth on their surfaces, AGBs retained diffusible bacterial metabolites and quorum-sensing molecules within the hydrogel matrix. Visible accumulation of bacterial pigments, together with increased retention of N-acyl-homoserine lactones in *P. aeruginosa* cultures, indicates that the hydrogel matrix can function as a local reservoir for small molecules [5]. Such chemically retentive behaviour may partially resemble hydrated host-associated microenvironments, including mucus layers, extracellular debris, or necrotic tissue material encountered during chronic infections [69]. The observed accumulation of pyoverdine-, pyocyanin-, and staphyloxanthin-associated metabolites further supports the concept that hydrogel substrates may influence local chemical interactions within biofilms.

The quantitative proteomic analyses performed in this study revealed pronounced substrate-dependent physiological differences between biofilms formed on AGBs and those formed on glass beads. Across all three bacterial species examined, biofilms cultivated on the hydrogel substrate exhibited distinct regulatory, metabolic, and virulence-associated expression profiles. In *E. coli*, these differences were primarily associated with ribosomal regulation and translational restructuring. In *P. aeruginosa*, AGB-associated biofilms showed increased abundance of extracellular virulence factors and pyoverdine-associated proteins, whereas glass-associated biofilms displayed broader stress-associated regulatory responses. *S. aureus* exhibited extensive substrate-dependent restructuring of regulatory pathways, stress adaptation, quorum-sensing-associated proteins, and virulence-associated factors. Together, these findings indicate that biofilms formed on hydrogels and abiotic substrates adopt distinct physiological states, supporting the idea that substrate composition substantially contributes to biofilm-associated bacterial physiology [70]. Glass bead systems have been widely adopted for their simplicity, reproducibility, and suitability for disinfectant testing [28]. However, the distinct physiological signatures observed in the present study suggest that substrate composition can influence biofilm-associated bacterial physiology beyond simple biomass formation. This observation is consistent with growing evidence that the physicochemical properties of *in vitro* biofilm models can influence bacterial physiology, gene expression, and antimicrobial susceptibility, highlighting the importance of selecting experimental systems that appropriately reflect the biological environment under investigation [8,13].

The AGB platform also proved suitable for functional screening approaches. Biofilm-deficient mutants of *E. coli* and *P. aeruginosa* exhibited significantly impaired biofilm formation on AGBs compared with their parental wild-type strains, demonstrating that the system can reliably detect phenotypic differences in biofilm formation under standard cultivation conditions. These observations are consistent with previous studies demonstrating the importance of type I pili for *E. coli* biofilm formation [59] and the contribution of stress-protective pathways to biofilm-associated fitness and adaptation in *P. aeruginosa* [60,71]. Combined with the ability to chemically functionalise the hydrogel surface, this feature may support future applications in genetic screening, anti-biofilm compound discovery, and experimental evolution studies investigating biofilm adaptation and antimicrobial tolerance.

Beyond its application as an *in vitro* biofilm model, the hydrogel-based nature of the AGB system may offer broader opportunities in bioengineering and biomaterials research. The combination of biocompatibility, structural stability, permeability, and chemical modifiability could support future adaptation of the system for biofabrication, 3D bioprinting, and investigations of biofilms at liquid-air interfaces. Similar hydrogel-based platforms have attracted increasing interest in engineered microbial systems, bioprinting applications, and clinically relevant infection models, highlighting the growing demand for experimentally tractable biomaterial substrates that more faithfully reproduce complex microbial microenvironments [13,[72], [73], [74]].

More broadly, our findings support the concept that hydrated, chemically interactive substrates can substantially influence bacterial physiology and may offer experimentally tractable alternatives to conventional abiotic biofilm surfaces. This view is consistent with the growing recognition that the physicochemical properties of experimental biofilm models can influence microbial behaviour and that more clinically relevant *in vitro* systems are needed to reproduce host-associated biofilms better [5,8,13,69,72].

## 4 Conclusions

In this study, we developed a crosslinked agarose-gelatine hydrogel bead platform as a biologically compatible substrate for investigating bacterial biofilms. The AGBs supported robust biofilm formation by *E. coli*, *P. aeruginosa*, and *S. aureus* while remaining compatible with a broad range of established analytical approaches, including fluorescence and confocal microscopy, scanning electron microscopy, crystal violet quantification, viable-cell enumeration, and quantitative proteomics.

Beyond supporting biofilm growth, the hydrogel matrix enabled the retention of diffusible bacterial metabolites and quorum-sensing molecules, the chemical functionalisation of the bead surface, and the detection of biofilm-deficient phenotypes. Quantitative proteomic analyses further demonstrated that biofilms formed on AGBs exhibit physiological and regulatory states distinct from those observed on conventional glass-bead biofilms, indicating that substrate composition can substantially influence biofilm-associated bacterial behaviour. By providing a hydrated, chemically interactive alternative to conventional glass-bead systems, AGBs complement existing biofilm models and expand the experimental toolbox available for investigating biofilm physiology, host-relevant microbial microenvironments and biofilm-material interactions.

## 5 Materials and Methods

**Generation of agarose-gelatine beads (AGBs).** To produce the bead substrate, 4 g each of agarose and gelatine were added to 200 ml of distilled water (final concentration of 2% w/v each) in a 400 ml bottle, then autoclaved for 20 min at 121°C and 1 atm. After sterilisation and throughout bead production, the agarose-gelatine mixture was maintained in liquid form at 65°C using moderate stirring on a heated stir plate. In a separate container, 500 mL of mineral oil (Merck, Germany) was placed in an ice-water bath. Before use, the mineral oil was degassed under vacuum to remove air bubbles. Two procedures were used to produce AGBs. In the first approach, approximately 40 μl of the hot agarose-gelatine mixture was dispensed into the cooled mineral oil using a multistep pipette (Eppendorf, Germany). To minimise bead deformation and droplet partitioning during solidification, the pipette tip was maintained at a height of approximately 1-2 cm above the oil surface (Fig. S1).

A second, higher-throughput approach used a P-1 peristaltic pump (Pharmacia Biotech, Sweden) to enable continuous, semi-automated bead generation (Fig. 1). A stainless-steel capillary (2 mm internal diameter) was attached to the end of the silicone tubing to improve droplet stability and dispensing precision. Two silicone tubes (3 mm diameter, 0.5 m length) were connected by a 20 cm metal tube of matching diameter. The flexible ends were attached to the peristaltic pump, while the metal ends extended into the substrate reservoir or above the mineral oil collection container, respectively. The peristaltic pump generated a stable flow of uniform agarose-gelatine droplets, which solidified upon entering the cooled mineral oil, producing spherical AGBs of ∼4 mm in diameter. Following production, beads were recovered by sieving and washed repeatedly with distilled water until residual mineral oil was removed. A 5 min resting period was included between washes to facilitate phase separation between the aqueous and oil fractions.

**Crosslinking of the AGBs.** Washed AGBs were crosslinked with divinyl sulfone (DVS) using a modified version of the method originally described by Porath, Låås, and Janson [18]. Beads were weighed to calculate the required DVS-to-gel ratio (v/w) (Merck, Germany). Crosslinking reactions were performed using approximately five volumes of 0.5 M sodium carbonate buffer (pH 11) per volume of gel beads. The beads were transferred into an 800 ml flask, and DVS was added to a final concentration of 0.5% (v/v). The reaction proceeded overnight at room temperature with gentle shaking, while the flask was sealed with parafilm to minimise evaporation.

Following crosslinking, the beads were washed extensively with distilled water until the pH reached neutrality. Residual reactive vinyl groups were subsequently quenched by incubation in five volumes of 0.5 M sodium carbonate buffer (pH 9) containing 0.5% beta-mercaptoethanol. The reaction proceeded overnight at room temperature with shaking in the dark. Afterwards, the beads were washed five times with distilled water. To further block remaining reactive groups, the beads were incubated overnight in five volumes of 1 M sodium carbonate buffer (pH 10) supplemented with 10% (w/v) glycine. The beads were subsequently washed three times with five volumes of 0.1 M carbonate-bicarbonate buffer (pH 10), followed by three washes with 0.1 M citrate buffer (pH 4). Finally, the beads were washed three additional times with distilled water and transferred directly into five volumes of bacterial growth medium, either lysogeny broth (LB) or Mueller-Hinton broth (MHB). The bead-containing media were autoclaved and stored at 4°C until use. A detailed step-by-step protocol is provided in Supplementary Protocol I.

**Microscopy of Bead Surfaces.** Intact surfaces of AGBs and glass beads were visualised using a VHX-X1 microscope (Keyence, Austria). Glass beads were placed directly into 45 mm Petri dishes for imaging. For AGB imaging, beads were removed from the preservation medium (LB), excess liquid was carefully pipetted off, and the beads were transferred into separate 45-mm Petri dishes before examination at different magnifications.

**Bacterial strains and cultivation conditions.** The bacterial strains used throughout this study included *E. coli* K-12 MG1655 [75]*, E*. *coli* BW25113 and its derivative mutant *E. coli* BW25113 *fimH::Kn* from the Keio collection [76], *P. aeruginosa* PAO1 [77], *P. aeruginosa* PA14 and its derivative mutant *P. aeruginosa PA14 pfpI::*MAR2xT7 [78], and *S. aureus* SH1000 [79].

Unless otherwise indicated, bacteria were cultivated from frozen glycerol stocks on lysogeny broth (LB) agar plates and incubated overnight at 37°C. Single colonies were inoculated into 10 ml LB broth in 50 ml Falcon tubes and cultured overnight at 37°C with shaking at 200 r.p.m. Cell density was estimated spectrophotometrically by measuring optical density at 600 nm (OD600). For biofilm inoculation, overnight cultures were diluted to approximately 10^6^ CFU/ml.

Biofilms were cultivated on AGBs or conventional glass beads in 24-well polypropylene microplates. Following inoculation, the culture was incubated with beads at 37°C under moderate agitation. For experiments extending beyond 24 h, the culture medium was replaced every 24 h. Detailed cultivation procedures are provided in Supplementary Protocol II.

Biofilm-associated cells were recovered from AGBs and glass beads using a combination of enzymatic digestion, sonication and vortexing, followed by serial dilution and CFU enumeration. Detailed recovery procedures are provided in Supplementary Protocol III.

**GFP-labelled strains.** For fluorescence microscopy experiments, GFP-expressing derivatives of *Escherichia coli* MG1655, *Pseudomonas aeruginosa* PAO1, and *Staphylococcus aureus* SH1000 were generated using previously described GFP-expression systems [[80], [81], [82]]. Plasmids pGRG36-Kn-PA1-GFP, pMF230, and pTH100 were obtained from Addgene, propagated in appropriate donor strains, and introduced into recipient strains by electroporation or triparental conjugation as previously described [81,83,84]. For *P. aeruginosa*, triparental conjugation was performed using *E. coli* S17-1 λpir as the helper strain carrying pRK2013. Transformants were selected using the appropriate antibiotics and verified by GFP fluorescence before storage at −80°C.

**Crystal violet staining.** Semi-quantitative assessment of total biofilm biomass was performed using crystal violet staining as previously described [63]. For substrate comparison experiments, 4 mm AGBs and 4 mm glass beads (Merck, Germany) were inoculated with *P. aeruginosa* PAO1 and *S. aureus* SH1000 biofilms in 24-well polypropylene plates (Carl Roth, Germany).

Following biofilm maturation and a final wash step, beads were covered with 500 μl of 0.9% NaCl solution and stained with an additional 500 μl of 0.1% crystal violet solution (Merck, Germany), prepared in saline, for 1 min. The staining solution was subsequently removed by aspiration, and the beads were rinsed repeatedly with 1 mL of distilled water prewarmed to 45°C. A 5 min incubation period was included between washes to facilitate the removal of background stain. Washing was continued until the rinse solution became visually clear or reached an OD592 below 0.01.

Crystal violet retained within the biofilm biomass was solubilised by adding 1 ml of a solution containing 95% ethanol [71] and 0.05% Triton X-100 [85]. Beads were incubated for 30 min at 45°C with gentle shaking. Subsequently, 200 μl of each sample was transferred into a 96-well plate, and crystal violet absorbance was quantified at 592 nm.

**Scanning electron microscopy (SEM).** Biofilm formation on AGB surfaces was visualised using SEM (EVO MA 10, Carl Zeiss, Germany). Before fixation, colonised beads were washed with saline as described above. Because of the toxicity of the fixation reagents, all subsequent preparation steps were performed under a safety cabinet.

Six mature biofilm samples of *E. coli* K-12, *P. aeruginosa* PAO1, and *S. aureus* SH1000 grown on AGBs were collected with sterile forceps and transferred into 10-ml glass jars. Primary fixation was performed by incubating samples in 5 ml of 2.5% glutaraldehyde (25% stock solution, Carl Roth, Germany) prepared in 0.05 M cacodylate buffer for 1 h. Following fixation, the solution was gradually replaced with fresh cacodylate buffer during subsequent preparation steps to prevent complete dehydration of the hydrogel beads. Samples were then washed three times with 0.05 M cacodylate buffer for 10 min each.

Secondary fixation and contrasting were performed using 2% osmium tetroxide (ReagentPlus®, 99.8%, Merck, Germany) in distilled water [86]. Following removal of the cacodylate buffer, 2 ml of osmium tetroxide solution was added to each sample and the samples were incubated for 30 min, followed by an additional cacodylate buffer wash.

Dehydration of fixed biofilm-containing AGBs was performed using a graded ethanol series prior to critical point drying with carbon dioxide (CO2). Samples were transferred into the chamber of a critical point dryer (Foissner vessel, CPD 030, BAL-TEC-Leica, Germany) and sequentially incubated for 10 min in 30%, 50%, 70%, 90%, and twice in 100% ethanol containing a molecular sieve.

During critical point drying, residual water and ethanol were replaced with liquid CO2. The transition of CO2 into the gaseous phase at critical temperature and pressure conditions preserved the hydrogel surface architecture while minimising structural damage associated with the liquid-gas transition during conventional drying.

Following drying, samples were mounted on 12 mm aluminium stubs and sputter-coated with gold using an SCD 040 coating system (BALZERS, Liechtenstein). Argon was used as the inert gas during coating to ensure homogeneous metal deposition. Samples were imaged at working distances of 6.5-12 mm and magnifications ranging from 50× to 27,000×.

**Confocal fluorescence microscopy (CLSM).** AGBs were initially cultivated with a GFP-expressing *E. coli* strain as described above. Following biofilm maturation and a final saline wash, the samples were transferred into glass-bottom chambered coverslips for confocal microscopy (μ-Slide 8 Well, Ibidi, Germany). Beads were submerged in saline, chambers were sealed with lids, and imaging was performed using an inverted confocal laser scanning microscope (SP8-1, Leica, Germany) equipped with a 40× objective. GFP fluorescence was excited at 488 nm and detected at 510 nm. Both single-plane images and Z-stacks were acquired from bead surfaces and cross-sections, and subsequently processed using ImageJ.

For live fluorescence microscopy, the same cultivation and preparation procedures were used for GFP-expressing *E. coli*, *P. aeruginosa*, and *S. aureus* strains with minor modifications. To stabilise 4 mm AGBs during microscopy, a custom 3D-printed adapter was developed for standard microscope coverslips (60 × 25 mm). The adapter was designed using AutoCAD and printed with a Form 4B 3D printer (Formlabs, USA). The structure measured 25 × 25 mm and contained a central 5 mm cavity designed to securely position individual AGBs during imaging (Fig. S4). The corresponding 3D_bead_adapter.stl file is provided as a supplementary file. The adapter enabled stable live imaging of *E. coli*, *P. aeruginosa*, and *S. aureus* biofilms using a DMi 8 inverted microscope (Leica, Germany) equipped with a 100× oil-immersion objective (Fig. 3B).

**Coupling of melittin to the AGBs.** Melittin was coupled to AGBs using a modified cyanogen bromide-based coupling protocol adapted from affinity chromatography procedures for crosslinked agarose matrices, as described by March et al. [62]. Washed AGBs, prepared as described above, were first activated with cyanogen bromide before ligand coupling.

Coupling reactions were performed using 500 μl coupling buffer (0.1 M sodium bicarbonate, pH 9.5) per bead together with the ligand of interest. Melittin (5 mg/ml; GenScript, USA) or bovine serum albumin (BSA) (1 mg/ml; Merck, Germany) was added to the activated beads, and the coupling reactions were carried out for 20 h at 4°C.

Following coupling, the beads were washed with 20 vol of buffer containing 0.1 M sodium acetate (pH 4), 2 M urea, and 0.1 M sodium bicarbonate (pH 10). Beads were subsequently dialysed against a 100-fold volume of sterile 0.9% NaCl solution and stored at 4°C until use.

All procedures were performed under sterile conditions in a laminar-flow hood using sterile filtered buffers to minimise contamination.

**Staphyloxanthin accumulation.** *S. aureus* SH1000 biofilms grown on AGBs or glass beads were cultivated in 2 ml M9 medium with moderate shaking at 37°C for 48 h, with medium replacement every 24 h. The M9 medium (Merck, Germany) was supplemented with 0.4% casamino acids (Carl Roth, Germany), 5 mM MgSO4 (Carl Roth, Germany), and 0.5% glucose as final concentrations. Glucose and MgSO4 were added after autoclaving.

Following cultivation, cultures were centrifuged for 1 min at 10,000 × g, and supernatants were discarded. Individual beads were washed twice with 1 ml 0.9% NaCl solution. Staphyloxanthin extraction was performed by incubating each bead in 500 μl of methanol at 55°C for 30 min. Samples were subsequently centrifuged for 2 min at 10,000 × g.

For spectrophotometric quantification, 300 μl of each extract was transferred into a polystyrene 96-well plate, and absorbance was measured at OD 450 using a GloMax Explorer plate reader (Promega, USA). Five biological replicates were analysed per condition.

To normalise pigment production relative to biofilm abundance, OD_450_ values were divided by the corresponding CFU counts for each bead. Staphyloxanthin production was therefore expressed as OD_450_/CFU ratios and subsequently compared between AGB and glass-bead biofilms.

**Quantification of the virulence factors pyocyanin and pyoverdine.** To investigate substrate-dependent differences in *P. aeruginosa* PAO1 virulence-associated metabolite production, bacterial supernatants were collected from six mature biofilm samples cultivated on AGBs or glass beads in 24-well plates. Supernatants from all wells belonging to the same condition were pooled, transferred into 15 ml Falcon tubes, and centrifuged at 4000 × g for 15 min at 20°C to remove bacterial cells.

Pyocyanin extraction was performed using a modified chloroform-based protocol. Briefly, 5 ml supernatant was mixed with 1 ml chloroform and incubated for 10 min with intermittent vortexing. Following phase separation, the lower organic phase containing pyocyanin was collected. After clarification by centrifugation for 1 min at 10,000 × g, 700 μl of the organic phase was transferred into fresh tubes and re-extracted using an equal volume of 0.1 N HCl. This produced an upper aqueous phase with characteristic red colouration.

For spectrophotometric quantification, seven 100 μl aliquots from each aqueous phase were transferred into polystyrene 96-well plates, and absorbance was measured at OD520 using 0.1 N HCl as the blank. Five biological replicates were analysed for each condition. To normalise pyocyanin production relative to biofilm abundance, absorbance values were divided by the average CFU counts of the corresponding experimental group.

Pyoverdine quantification was performed using the same cultivation setup and replicate number as described for pyocyanin analysis. Supernatants from 24 h biofilm cultures were centrifuged at 10,000 × g for 10 min, and pyoverdine-associated fluorescence was measured with excitation at 405 nm and emission at 450 nm.

**AHL level determination**. N-acyl-homoserine lactone (AHL) activity in *P. aeruginosa* PAO1 cultures was assessed using a previously described colourimetric assay [65]. Briefly, bacterial cultures were grown overnight in 5 ml Mueller-Hinton broth (Merck, Germany) at 37°C. Subsequently, 1.5 ml aliquots were centrifuged at 10,000 × g for 15 min, and supernatants were collected following repeated clarification steps to remove residual bacterial cells. Clarified supernatants were filtered through 0.2 μm membrane filters (Sartorius, Germany).

AHL-containing compounds were extracted from the filtrates using ethyl acetate by liquid-liquid extraction [87]. Following mixing and phase separation, the upper organic phase was collected, while the aqueous phase underwent two additional extraction cycles. Organic fractions from the same sample were pooled and dried at 40°C.

Dried extracts were resuspended, and 40 μl aliquots were transferred into 96-well polystyrene plates. For colourimetric detection, 50 μl of a 1:1 mixture of hydroxylamine (2 M) and NaOH (3.5 M) was added to each well, followed by an equal volume of ferric chloride solution (10% ferric chloride in 4 M HCl) mixed 1:1 with 95% ethanol. Absorbance was subsequently measured at OD520.

Dried AHL extracts were stored at −20°C for further analysis. The development of a dark brown colouration indicated the presence of lactone-containing compounds. Because AHLs are susceptible to alkaline hydrolysis via pH-dependent lactonolysis, acidic conditions were monitored and maintained throughout the extraction procedure to preserve the integrity of the lactone ring.

**Agarose-gelatine stiffness measurement**. The mechanical stiffness of AGBs, with or without DVS crosslinking, was determined using a static compression assay described previously [88]. Beads with a diameter of approximately 4 mm were equilibrated overnight at room temperature before mechanical testing. Young's modulus values were calculated by fitting the linear region of the resulting stress-strain curves. Five independent samples were analysed for each condition.

**Sample preparation for bacterial biofilm proteomics.** Each experimental condition consisted of six biological replicates. Samples consisted of three colonised beads from glass, agarose-gelatine, or polystyrene substrates collected from multiwell plates. For protein extraction, 500 μl denaturation buffer (6 M urea, 2 M thiourea in 10 mM HEPES, pH 8.0) was added to each sample. Samples underwent five freeze-thaw cycles to promote bacterial lysis and protein release.

Protein digestion was subsequently performed in solution as previously described [89]. Briefly, proteins suspended in denaturation buffer were reduced with 10 mM dithiothreitol (DTT) in 50 mM ammonium bicarbonate (ABC) for 30 min, then alkylated with 55 mM iodoacetamide for 20 min. After alkylation, samples were protected from light until digestion was terminated.

Initial protein digestion was performed using Lysyl endopeptidase (LysC, Wako, Japan) diluted in 50 mM ABC and added at a ratio of 1 μg enzyme per 50 μg protein. Samples were incubated for 3 h at room temperature. Following LysC pre-digestion, samples were diluted fourfold with 50 mM ABC and subjected to overnight trypsin digestion using sequencing-grade modified trypsin (Promega, USA) at 1 μg per reaction.

Digestion reactions were terminated by acidification with 5% acetonitrile and 0.3% trifluoroacetic acid (final concentrations). Peptides were subsequently purified and concentrated using the StageTip protocol [89] and the eluates were vacuum-dried before downstream proteomic analysis.

A detailed step-by-step protocol is provided in Supplementary Protocol IV.

**Nano liquid chromatography-mass spectrometry (LC-MS) and data analysis.** Peptides were reconstituted in 30 μl solution containing 0.05% trifluoroacetic acid and 4% acetonitrile in water. Subsequently, 2 μl of each sample was analysed using an Ultimate 3000 reversed-phase capillary nano liquid chromatography system coupled to a Q Exactive HF mass spectrometer (Thermo Fisher Scientific).

Samples were initially loaded onto a trap column (PepMap100C18, 3 μm, 100 Å, 75 μm internal diameter × 2 cm; Thermo Fisher Scientific) equilibrated with 0.05% trifluoroacetic acid in water. Following inline switching of the trap column, peptide separation was performed using a capillary analytical column (Acclaim PepMap100C18, 2 μm, 100 Å, 75 μm internal diameter × 25 cm; Thermo Fisher Scientific) at a flow rate of 300 nl/min.

Mobile phase A consisted of 0.1% formic acid in water, while mobile phase B contained 0.1% formic acid in 80% acetonitrile and 20% water. Columns were pre-equilibrated with 5% mobile phase B, followed by a linear gradient from 5% to 44% mobile phase B over 35 min.

Mass spectra were acquired in data-dependent acquisition mode using a full MS survey scan range of *m*/*z* 300-1650 at 60,000 resolution, followed by MS/MS analysis of the 15 most intense precursor ions at 15,000 resolution. Dynamic exclusion was set to 20 s. Automatic gain control values were set to 3 × 10^6 for MS scans and 1 × 10^5 for MS/MS scans.

Raw MS and MS/MS data were analysed using MaxQuant software (version 2.0.3.0) with the integrated Andromeda search engine [90]. Spectra were searched against UniProt reference proteomes for *E. coli* (4448 proteins; Proteome ID UP000000625), *P. aeruginosa* (5564 proteins; Proteome ID UP000002438), and *S. aureus* (2889 proteins; Proteome ID UP000008816).

Filtering and statistical analyses were performed using Perseus software (version 1.6.14) [91]. Missing values were imputed using default settings. Mean log2 LFQ intensity differences between experimental groups (glass beads versus AGBs) were calculated using Student's t-tests with false discovery rate (FDR) correction at a threshold of 0.05.

**Statistical analysis.** Statistical analyses were performed using GraphPad Prism (version 9) and R version 4.3.3 [92]. Pairwise comparisons were analysed using Student's t-test when data were normally distributed and exhibited homogeneous variance. For non-parametric datasets, the Mann-Whitney *U* test was used instead. Multiple-group comparisons were performed using one-way ANOVA, followed by the Holm-Šídák post hoc correction for parametric data, or the Kruskal-Wallis test for non-parametric data.

## Funding

This research was supported by the VolkswagenStiftung as part of a multi-institutional project (project no. 0065316), supporting DR and JR. We also thank the OneHealth PhD program funded by Vetmeduni, WWTF Vienna to ARR, the program Erasmus-Mundus-Master to NP.

## CRediT authorship contribution statement

**Dan Roizman:** Conceptualization, Data curation, Formal analysis, Investigation, Methodology, Writing – original draft. **Maren Herzog:** Conceptualization, Data curation, Formal analysis, Investigation, Methodology. **Arpita Nath:** Data curation, Formal analysis, Investigation, Methodology, Software. **Nivetha Pachaimuthu:** Investigation, Methodology. **Ahmad Hujeirat:** Investigation, Methodology. **Benno Kuropka:** Data curation, Formal analysis, Investigation, Methodology, Writing – review & editing. **Jens Rolff:** Conceptualization, Funding acquisition, Investigation, Project administration, Resources, Supervision, Writing – review & editing. **Alexandro Rodríguez-Rojas:** Conceptualization, Data curation, Formal analysis, Funding acquisition, Investigation, Methodology, Project administration, Resources, Supervision, Writing – original draft, Writing – review & editing.

## Competing interests statement

The authors declare no competing interests.

## Data Availability

The datasets generated and analysed in this study, including raw data files, are available in the manuscript and the supplementary information files.

## Abbreviations

DVS, divinyl sulfone; AGBs, agarose-gelatine beads; CFU, colony-forming units; SEM, scanning electron microscopy; LC-MS, liquid chromatography-mass spectrometry; OD, optical density; GFP, green fluorescent protein.
