# Supplementary material for: Crosslinked agarose-gelatine beads as a substrate for investigating biofilms of bacterial pathogens
Source: Biofilm. 2026 Jul 8;12:100380. doi: 10.1016/j.bioflm.2026.100380 (PMC13382339; doi:10.1016/j.bioflm.2026.100380)
Supplement: Multimedia component 4 [file mmc4.docx]

**Supplementary material**

**Crosslinked agarose-gelatine beads as a substrate for investigating biofilms of bacterial pathogens**

Dan Roizman^1§^, Maren Herzog^1§^, Arpita Nath^1^, Nivetha Pachaimuthu^2^, Ahmad Hujeirat^1^, Benno Kuropka^3^, Jens Rolff^1*^, Alexandro Rodríguez-Rojas^1,2*^


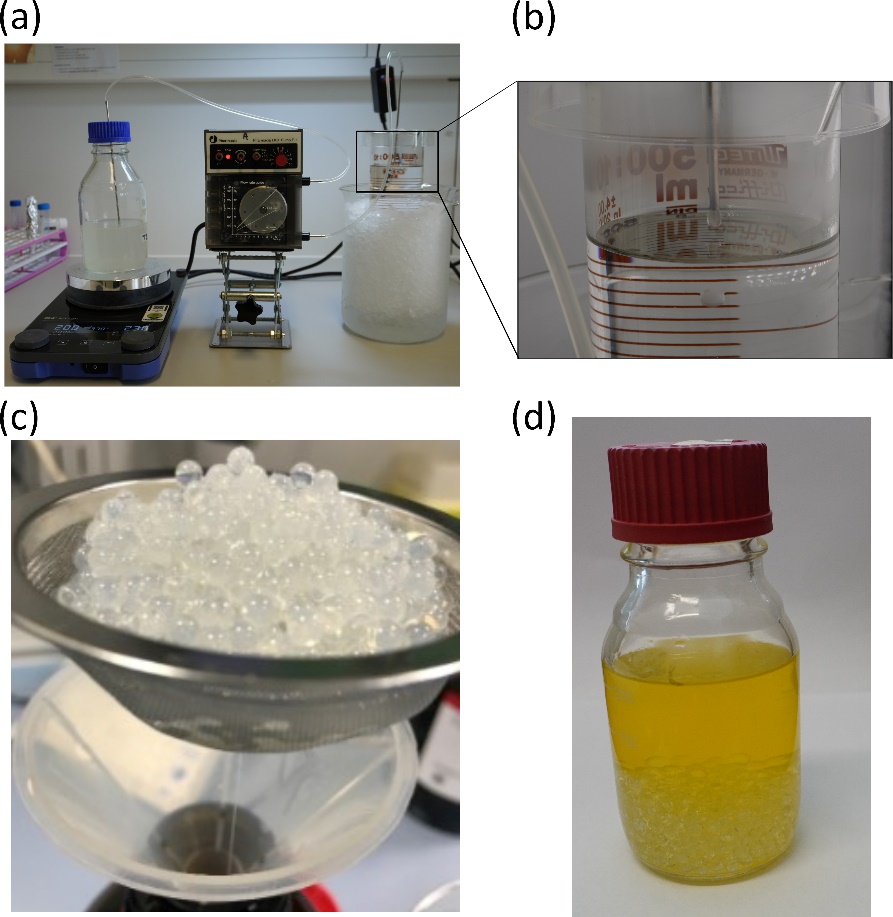


**Figure S1.** Production process for the agarose-gelatine beads. (A) The bead substrate is carefully placed on a heating plate, with continuous stirring, ensuring that the temperature is maintained, and that the bottle is positioned above the pump level and the collection vessel in the ice container. (B) Droplets form at the end of the metal tube according to the pump flow rate. The beads gain their spherical form due to hydrogel surface tension, and solidify due to the drastic drop in temperature, and the inert oil's hydrophobic environment. (C) The resulting beads are sieved and washed with tap water until it flows transparently, with a household detergent added to the first wash to enhance the displacement of oil molecules. The mineral oil is filtered for reuse. The beads are then ready for the following crosslinking steps. (D) The resulting beads, in the medium of choice following autoclaving, can be stored for at least several months at 4°C until required. For visualisation, refer also to supplementary video S1.


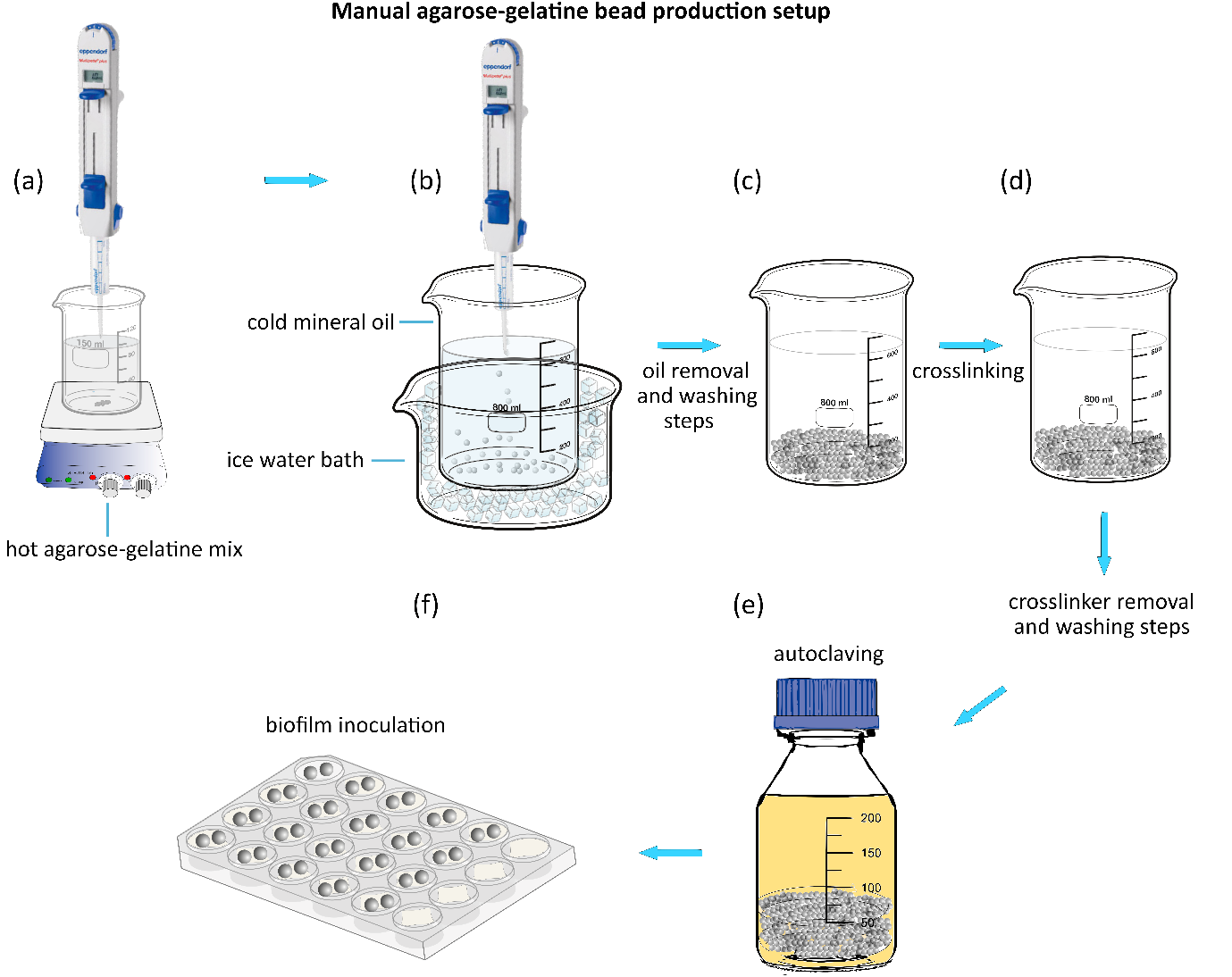


**Figure S2**. This figure illustrates a more accessible setup for bead production using a multi-step pipette instead of a peristaltic pump, as shown in Fig. 1. In the initial step (a), a molten agarose-gelatine mixture (2% each in distilled water) is prepared and placed on a magnetic stirrer with a hot plate to maintain the mixture in a molten state. This solution is continuously added to ice-cold mineral oil using a multi-step pipette for a fast manual operation, followed by the same steps as described in Fig. 1. In step (b), the mineral oil is removed, and washing steps, including the use of detergent, are performed to eliminate oil residues. Following this, in step (c), the beads undergo crosslinking using divinyl sulfone (DVS), followed by washing to remove residual crosslinker. After removing the crosslinker, the beads are transferred to a bottle and autoclaved at 121°C for 15 minutes (d), a critical step that ensures the long shelf life of the beads. Once autoclaved, the beads can be stored for several months until use. In the final step (e), the beads are transferred to a 24-well plate, where the chosen bacteria are inoculated, and the biofilm is established, as previously described. For additional details, see the Materials and Methods section and supplementary video S2.


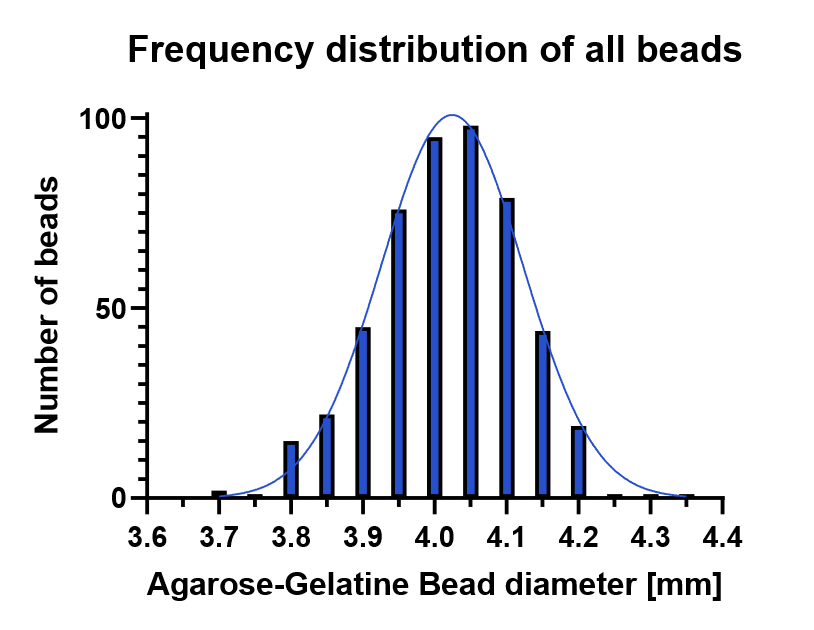

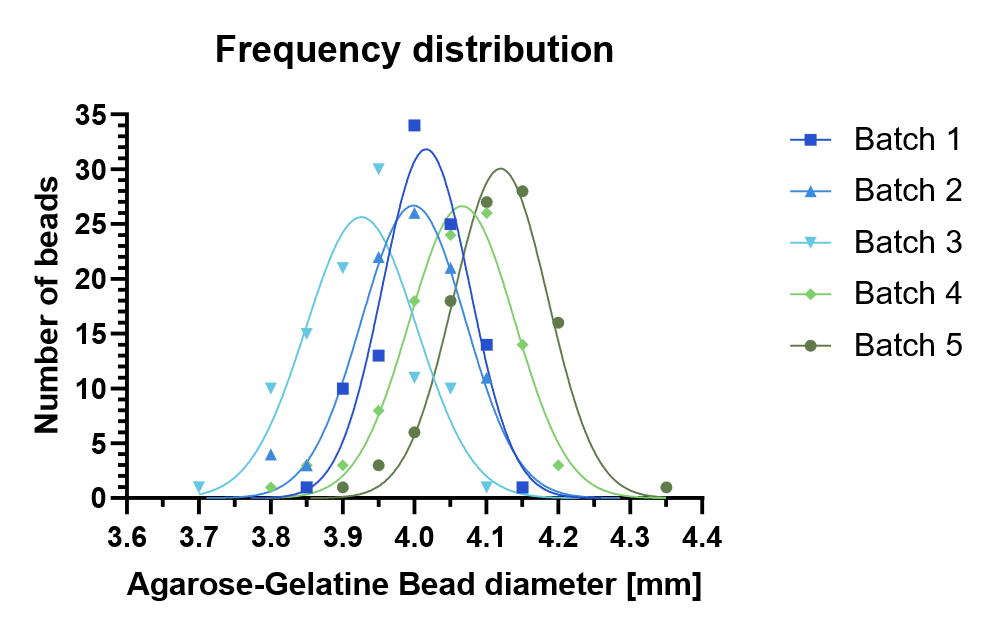


**Figure S3. Statistical validation of the consistent size distribution of five independent batches of agarose-gelatine bead samples (N=100).** (A) The bead population size consistently followed a normal distribution as per the D'Agostino & Pearson test with *p*-values above α=0.05. The data plotted here is the number of beads per 0.05 mm for simplification purposes. Means range between 4.11-3.92 mm with standard deviations below 0.08 mm. The frequency distribution of the whole sampled population of agarose-gelatine beads (B) 99% of the produced beads follow a normal distribution as represented by the Gaussian curve (B).


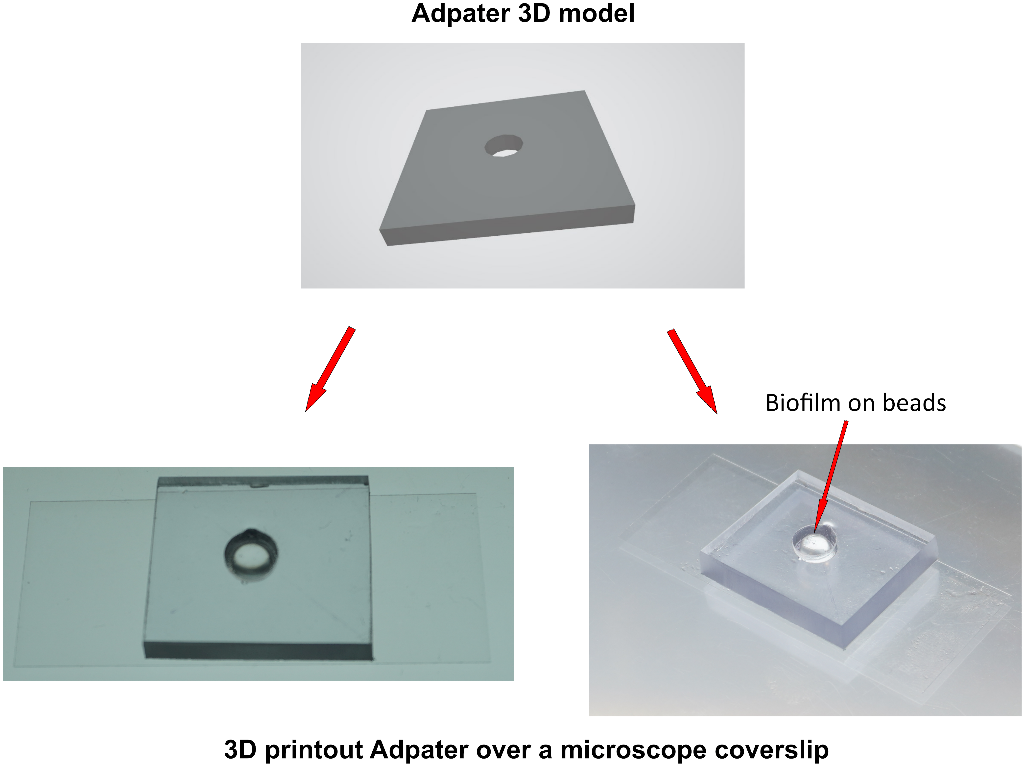


**Figure S4.** A square 3D printed adapter, measuring 25 mm x 25 mm and containing a central hole of 5 mm diameter, was designed in AutoCAD and printed to provide a stable platform for microscopy of biofilms grown on beads. Biofilms formed on 4 mm beads were positioned within the central opening, as indicated by the red arrow. A coverslip was attached to the underside using optical adhesive, providing a flat surface for stable positioning of the samples. The device is compatible with 100× oil immersion objectives. The file 3D_bead_adapter.stl is provided as a supplementary file.

**S1 Protocol. Preparation of crosslinked agarose-gelatine beads (AGBs)**

**Materials**

- Agarose
- Gelatine
- Distilled water
- Mineral oil
- 400 mL glass bottle
- 1 L glass beaker
- Ice-water bath
- Peristaltic pump
- Silicone tubing
- Metal tubing
- Vacuum pump
- 0.5 M sodium carbonate buffer
- 1 N sodium hydroxide
- Divinyl sulfone (DVS)
- 2-mercaptoethanol
- Glycine
- 0.1 M carbonate-bicarbonate buffer, pH 10.0
- 0.1 M citrate buffer, pH 4.0, containing sodium chloride
- Parafilm
- Aluminium foil
- Shaker
- LB or Mueller-Hinton broth

**Procedure**

**Preparation of the agarose-gelatine mixture**

1. Add 4 g agarose and 4 g gelatine to 200 mL cold distilled water in a 400 mL glass bottle.
2. Autoclave the suspension at 121°C for 20 min.
3. Maintain the molten mixture at 60°C with continuous stirring throughout bead production.
4. Remove air bubbles under vacuum, if required.

**Formation of the beads**

1. Add approximately 500 mL mineral oil to a 1 L glass beaker.
2. Place the beaker in an ice-water bath to cool the oil.
3. Connect the peristaltic pump using silicone and metal tubing.
4. Position the tubing outlet approximately 1 to 2 cm above the oil surface.
5. Dispense the hot agarose-gelatine mixture into the cooled oil.
6. Adjust the flow rate to produce droplets of approximately 40 µL.
7. Collect 10 to 20 test beads and confirm that the diameter is approximately 4 mm.
8. Readjust the flow rate if necessary.
9. Continue dispensing until the required number of beads has been produced.

**Washing and recovery of the beads**

1. Collect the beads by sieving.
2. Wash the beads thoroughly with tap water until the wash solution runs clear.
3. Recover and filter the mineral oil for reuse, if required.

**Crosslinking**

1. Weigh the washed beads.
2. Transfer the beads to approximately 550 mL of 0.5 M sodium carbonate buffer.
3. Adjust the buffer to pH 11.0 with 1 N sodium hydroxide.
4. Add DVS at 1.25 mL per 250 g wet beads.
5. Seal the vessel with parafilm.
6. Incubate the suspension overnight at room temperature with shaking.

**Washing after crosslinking**

1. Remove the crosslinking solution.
2. Wash the beads repeatedly with distilled water.
3. Allow the beads to rest for at least 10 min between washes.
4. Continue washing until the wash solution reaches neutral pH.

**Quenching and removal of residual reactive groups**

1. Suspend the beads in a neutral or slightly alkaline medium.
2. Add 2-mercaptoethanol at 0.01 mL per mL of bead suspension.
3. Cover the vessel with parafilm and aluminium foil.
4. Incubate the suspension overnight at room temperature with shaking.
5. Remove the solution and wash the beads several times with distilled water.
6. Suspend the beads in 10% glycine prepared in 1 M sodium carbonate buffer, pH 10.0.
7. Incubate overnight at room temperature with shaking.
8. Wash the beads three times with 0.1 M carbonate-bicarbonate buffer, pH 10.0.
9. Wash the beads with 0.1 M citrate buffer, pH 4.0, containing sodium chloride.

**Sterilisation and storage**

1. Transfer the beads into the required storage medium, such as LB or Mueller-Hinton broth.
2. Autoclave the beads in the storage medium.
3. Store the beads at 4°C until use.

**Notes**

- Keep the beads hydrated throughout preparation, processing, sterilisation, and storage.
- Do not allow the beads to dry.
- Do not desiccate and rehydrate the beads.

**S2 Protocol. Growth of bacterial biofilms on agarose-gelatine beads**

**Materials**

- Sterile agarose-gelatine beads
- Bacterial overnight culture
- Appropriate growth medium
- 24-well microplate
- 0.9% NaCl solution
- Pipettes and sterile tips
- Vacuum aspirator
- Orbital shaker

**Procedure**

**Preparation of the inoculum**

1. Prepare an overnight culture of the bacterial strain in the appropriate growth medium.
2. Dilute the overnight culture to approximately 1 x 10^6 CFU/mL.

**Inoculation of the beads**

1. Place two sterile agarose-gelatine beads into each well of a 24-well microplate.
2. Add 1 mL of the diluted bacterial suspension to each well.

**Biofilm growth**

1. Incubate the plate at 37°C on an orbital shaker at 20 to 30 rpm.
2. Incubate for the required period according to the bacterial species and experimental aim.

**Washing of the beads**

1. Remove the liquid phase carefully.
2. Wash the beads three times with 0.9% NaCl by gently adding and removing the solution with a pipette.
3. Use a vacuum aspirator if needed to improve removal of residual liquid.

**Preparation for downstream analysis**

1. At the end of the incubation period, wash the beads again as described above.
2. Transfer the washed beads to a fresh plate or tube for downstream analyses.

**Notes**

- Use the same incubation time for all comparable experimental groups.
- Use the same washing procedure for all samples within an experiment.
- Specify the incubation time for each bacterial strain in the main Methods section.

**S3 Protocol. Extraction of viable bacteria from biofilms grown on agarose-gelatine beads**

**Materials**

- Biofilm-containing agarose-gelatine beads
- 0.9% NaCl solution
- 2 mL microcentrifuge tubes
- Digestion buffer containing 150 mM NaCl, 50 mM Tris, 10 mM MgCl2, 5 mM L-cysteine, 5 mg/mL DNase I, and 2 mg/mL RNase A, pH 7.5
- Papain stock solution, 10 mg/mL
- Thermoblock or water bath set to 37°C
- Sonication bath
- Microcentrifuge
- Selective agar plates

**Procedure**

**Sample collection**

1. Grow biofilms on agarose-gelatine beads according to S2 Protocol.
2. Include treated and untreated samples as required.
3. Wash the beads with 0.9% NaCl.
4. Transfer two beads from each sample into a separate 2 mL microcentrifuge tube.

**Enzymatic digestion**

1. Add 200 µL of digestion buffer to each tube.
2. Incubate the tubes for 15 min at 37°C.
3. Mix the tubes intermittently during incubation.

**Papain treatment and sonication**

1. Add 20 µL of papain stock solution to each tube.
2. Place the tubes in a pre-warmed sonication bath.
3. Sonicate for 5 min.

**Recovery of bacterial cells**

1. Transfer the supernatant to a fresh tube.
2. Wash the beads once with 1 mL of digestion buffer.
3. Combine this wash with the first supernatant.
4. Centrifuge the combined suspension at 10,000 x g for 5 min.
5. Remove the supernatant.
6. Resuspend the pellet in 1 mL of 0.9% NaCl.

**CFU determination**

1. Prepare serial dilutions as required.
2. Plate the dilutions on selective agar.
3. Incubate the plates under the appropriate conditions.
4. Count colonies and calculate CFU.

**Notes**

- Apply the same extraction procedure to all samples within an experiment.
- Report clearly how CFU values were normalised.

**S4 Protocol. Preparation of biofilm lysates from agarose-gelatine beads for proteomic analysis**

**Materials**

- Biofilm-containing agarose-gelatine beads
- 1.5 mL microcentrifuge tubes
- Dry ice or liquid nitrogen
- Water bath set to 37°C
- Microcentrifuge
- Protein assay reagents
- Urea buffer containing 6 M urea, 2 M thiourea, and 10 mM HEPES, pH 8.0
- DTT stock solution
- Iodoacetamide solution, 55 mM, freshly prepared
- 50 mM ammonium bicarbonate, freshly prepared
- Sequencing grade trypsin
- 10% trifluoroacetic acid
- Acetonitrile

**Procedure**

**Collection of biofilm material**

1. Grow biofilms on agarose-gelatine beads according to S2 Protocol.
2. Prepare at least five biological replicates per condition.
3. Wash the beads with 0.9% NaCl.
4. Transfer two beads from each sample into a separate 1.5 mL microcentrifuge tube.

**Extraction by freeze-thaw cycles**

1. Add 200 µL of urea buffer to each tube.
2. Freeze the samples on dry ice or in liquid nitrogen for at least 2 min.
3. Thaw the samples immediately in a 37°C water bath.
4. Mix gently by flicking the tube.
5. Repeat the freeze-thaw cycle four additional times, for a total of five cycles.

**Collection of crude lysate**

1. Centrifuge the samples at 10,000 x g for 10 min.
2. Transfer 100 µL of the supernatant to a fresh tube.
3. Keep the lysates on ice.
4. Measure the total protein concentration using an appropriate protein assay.
5. Use approximately 50 µg of total protein for digestion.

**Reduction and alkylation**

1. Adjust each sample containing 50 µg protein to a final volume of 50 µL with urea buffer.
2. Add 2.5 µL DTT solution.
3. Incubate for 30 min at room temperature.
4. Add 2.5 µL of freshly prepared 55 mM iodoacetamide.
5. Incubate for 30 min at room temperature in the dark.

**Enzymatic digestion**

1. Add 200 µL of freshly prepared 50 mM ammonium bicarbonate to reduce the urea concentration to below 2 M.
2. Add 2 µL of sequencing grade trypsin at 0.5 µg/µL.
3. Incubate overnight at room temperature.

**Termination of digestion**

1. Add 8 µL of 10% trifluoroacetic acid.
2. Mix by pipetting.
3. Add 14 µL acetonitrile.

**Notes**

- Prepare fresh reagents where indicated.
- Protect iodoacetamide from light.
- Minimise contamination during sample handling.

**S5 Protocol. Desalting of peptide samples using StageTips**

**Materials**

- Digested peptide samples
- 200 µL pipette tips
- Two C18 discs per StageTip
- Methanol
- Buffer A, 5% acetonitrile and 0.3% trifluoroacetic acid
- Buffer B, 80% acetonitrile and 0.3% trifluoroacetic acid
- 2 mL collection tubes
- Microcentrifuge
- Vacuum concentrator
- LC-MS sample buffer, for example 4% acetonitrile and 0.05% trifluoroacetic acid

**Procedure**

**Preparation of StageTips**

1. Prepare one StageTip per sample using a 200 µL pipette tip containing two stacked C18 discs.
2. Place each StageTip into a 2 mL collection tube.

**Activation and equilibration**

1. Add 100 µL methanol to each StageTip.
2. Centrifuge for 10 s at 10,000 x g.
3. Confirm that some methanol remains on the membrane.
4. If all solvent passes through immediately, replace the tip and prepare a new StageTip.
5. Centrifuge for 15 s at 3,000 x g to remove the methanol.
6. Add 200 µL Buffer A to each StageTip.
7. Centrifuge at 3,000 x g for 5 min.

**Sample loading and washing**

1. Load the acidified peptide sample onto the corresponding StageTip.
2. Centrifuge at 3,000 x g for 5 min.
3. Add 200 µL Buffer A.
4. Centrifuge again at 3,000 x g for 5 min.

**Elution and drying**

1. Place each StageTip into a clean collection tube.
2. Elute peptides with 100 µL Buffer B.
3. Dry the eluate in a vacuum concentrator.
4. Resuspend the peptides in a suitable LC-MS sample buffer, for example 20 µL of 4% acetonitrile and 0.05% trifluoroacetic acid.

**Notes**

- Use clean tubes and tips throughout.
- Avoid prolonged storage of peptide samples before LC-MS analysis.

**S6 Protocol. Crystal violet staining and quantification of biofilms grown on agarose-gelatine beads**

**Materials**

- Biofilm-containing agarose-gelatine beads
- Uninoculated agarose-gelatine beads for blank controls
- 24-well microplate
- 96-well microplate
- 0.9% NaCl solution
- Crystal violet solution, 0.05% in 0.9% NaCl
- 95% ethanol
- Triton X-100
- Water pre-warmed to 45°C
- Pipettes and sterile tips
- Vacuum aspirator
- Orbital shaker
- Plate reader

**Procedure**

**Preparation of samples**

1. Grow biofilms on agarose-gelatine beads according to S2 Protocol.
2. Include uninoculated beads as blank controls.
3. Wash the beads twice with 0.9% NaCl.

**Crystal violet staining**

1. Transfer the beads to a fresh 24-well plate if required.
2. Add 1 mL of 0.05% crystal violet solution to each well.
3. Incubate for 1 min.
4. Remove the staining solution using a pipette or vacuum aspirator.

**Washing**

1. Wash the beads several times with water pre-warmed to 45°C.
2. Allow the beads to stand for 10 min until no visible colour remains in the blank control beads.
3. Remove the wash solution.

**Drying and dye extraction**

1. Air-dry the beads at room temperature for 10 min.
2. Add 1 mL of 95% ethanol containing 0.05% Triton X-100 to each well.
3. Incubate for 30 min at 45°C on an orbital shaker.

**Measurement**

1. Transfer 200 µL from each sample to a 96-well plate.
2. Measure absorbance at 590 nm.

**Notes**

- Use blank beads for background subtraction.
- Expect a relatively high background signal because crystal violet can diffuse into the hydrogel matrix.
- Apply the same staining and washing procedure to all samples within an experiment.
